# Incidence of post-traumatic osteoarthritis in 44B ankle fractures: Analysis of risk factors

**DOI:** 10.1016/j.ocarto.2024.100507

**Published:** 2024-08-03

**Authors:** G. Caruso, E. Gambuti, A. Saracco, N. Biagi, E. Spadoni, L. Vigliaroli, L. Massari

**Affiliations:** a Department of Neurosciences and Rehabilitation, University of Ferrara, Via Aldo Moro 8, 44124, Ferrara, Italy; b Henley Business School, Business Informatics System and Accounting, Informatics Research Centre, University of Reading, UK; c Orthopaedic and Traumatology Unit, S. Anna University Hospital of Ferrara, Ferrara, Italy

**Keywords:** Ankle fractures, Malleolar fractures, Ankle osteoarthritis

## Abstract

**Objective:**

The purpose of this study was to analyse the clinical and radiographic data of a consecutive series of patients treated surgically for AO/OTA 44B ankle fracture at Ferrara University Hospital, Italy, with a view to identifying risk factors contributing to worse clinical and radiographic outcomes with a minium follow up of 6 years.

**Materials and methods:**

For each patient the following data were recorded: gender, age, Body Mass Index (BMI), follow up (months), previous ankle sprains, type of work, Kellgren-Lawrence (K&L) score, AO/OTA classification for ankle fracture, Foot and Ankle Disability Index (FADI score), ankle dislocation, syndesmotic transfixation, quality of reduction.

**Results:**

FADI score in patients with AO/OTA 44B1 fracture was 95.5±7.5, in 44B2 it was 90.0±8.4 and in 44B3 it was 84.0±13.0 (p25 it was 88.6±11.4 (p=0.047 95%I.C. 0.01-8.10). In case of fracture-dislocation there was a statistically significant difference in the FADI (94.4±6.0 vs 85.8±11.98)(P=0.002 95% I.C. 0.01-8.9). In the former group, there was a statistically significant difference in the ​the K&L (1.97±0.65 vs 2.63±0.85) (P=0.006 95% I.C 0.01-1.00).

Finally, the quality of the reduction was a statistically significant parameter in both the FADI and K&L (P=0.012 95% I.C. 0.90-10.60 and P=0.012 95%I.C. 0.01-1.00 respectively).

**Conclusion:**

The most influential risk factors for worse outcome in AO/OTA 44B ankle fractures were found to be BMI, injury severity, fracture-dislocation and reduction quality.

## 1 Introduction

Ankle osteoarthritis (AO) affects about 1% of the adult population and has a considerable impact on quality of life. It has an estimated prevalence of 30 cases per 100,000 inhabitants, and accounts for 3% of all patients with osteoarthritis [1]. The main symptoms, although non-specific, are pain, stiffness and joint swelling [2]. The major risk factors are trauma, inflammatory diseases (e.g., rheumatoid arthritis), overload and instability [3]. Of these, trauma is the most influential risk factor, causing about 70–80% of AO cases [4,5]. Such patients present advanced AO about 14 years earlier than patients with non-traumatic AO [5]. While the ankle joint has high articular congruence with thin cartilage and better repair capacity than the hip or knee [6], trauma results in alterations of the joint surface and contributes to a change in load transmission at the joint level [7].

Currently, fracture reduction is the only way of improving the outcome [8]. However, few studies have evaluated the risk factors for the development of osteoarthritis in patients with ankle fractures, and most have a small statistical sample [4] or focus on different trauma mechanisms. Hence, the purpose of this study was to analyse the clinical and radiographic data of a consecutive series of patients treated surgically for AO/OTA 44B ankle fracture at Ferrara University Hospital, Italy, with a view to identifying risk factors contributing to worse clinical and radiographic outcomes. The minimum follow-up was 6 years.

## 2 Materials and methods

This is a retrospective study based on analysis of data pertaining to a consecutive cohort of patients treated surgically for *trans*-syndesmotic AO/OTA 44B ankle fractures at the University of Ferrara (Azienda Ospedaliera Universitaria Sant'anna) Orthopaedic Operating Unit, Italy, between January 2012 and December 2016. The exclusion criteria were as follows: ankle fractures involving distal tibial diaphysis or tibial plafond; fibular fixation with intramedullary nails or other devices; age <18 years; pathological fractures induced by tumours or metastatic lesions; poor-quality x-rays; and follow up of less than 6 years.

For each patient the following data were recorded: gender, age, Body Mass Index (BMI), follow up (months), preoperative anaesthesiologic risk score (American Society of Anesthesiologists - ASA - score), previous emergency department visits for ankle sprains, type of work, Charlson Comorbidity index score (CCI score), Kellgren-Lawrence (K&L) score and AO/OTA classification for ankle fracture, Foot and Ankle Disability Index (FADI score), ankle dislocation, syndesmotic transfixation with screws, average length of stay, surgery within 3 days, quality of reduction. Joint reduction was assessed via postoperative step-offs in the articular surface, dime sign, Menard–Shenton line, tibiofibular clear space and medial clear spaceMeeting any one of these criteria prompted the classification “poor joint reduction”.

The K&L score is a common method of classifying the severity of osteoarthritis. It has five grades: grade 0, definite absence of x-ray changes of osteoarthritis; grade 1, doubtful joint space narrowing and possible osteophytic lipping; grade 2, definite osteophytes and possible joint space narrowing; grade 3, moderate multiple osteophytes, definite narrowing of joint space and some sclerosis and possible deformity of bone ends; grade 4, large osteophytes, marked narrowing of joint space, severe sclerosis and definite deformity of bone ends.

Joint function can be assessed via the region-specific self-report FADI score, whose 34 items are each scored on a 5-point Likert scale from 0 (unable to do) to 4 (no difficulty at all). The 4 pain items in the FADI are scored 0 (none) to 4 (unbearable). The FADI has a maximum score of 104 points.

According to the AO/OTA classification, *trans*-syndesmotic ankle fractures are denoted 44B. These fractures are further divided into: 44B1 (isolated *trans*-syndesmotic fracture of the fibula); 44B2 (*trans*-syndesmotic fracture of the fibula with medial lesion); and 44B3 (*trans*-syndesmotic fracture of the fibula with medial lesion and posterolateral rim fracture (Volkmann).

For posterior malleolus fractures, Bartonicek's classification was used [9]. The medial injuries encountered were medial malleolus fractures and deltoid ligament injuries. These were not considered separately in the statistical analysis in this study. All fractures were classed as AO/OTA 44B1, 44B2 or 44B3 by an orthopaedic surgeon experienced in foot and ankle surgery (C.G.). All patients considered in this study underwent a weight-bearing X-ray of the ankle. The radiographic images were analysed by an orthopaedic surgeon experienced in foot and ankle surgery, who provided the K&L score (L.M.) with the aid of Carestream Vue Picture Archiving and Communication system (PACs, version 12.2.5.00397) software. AO was defined by a K&L score greater than 2, and a FADI score was calculated for all patients.

## 3 Statistical analysis

Statistical analyses were conducted using MedCalc Statistical software. In all tests, statistical significance was set at P ​< ​0.05. Categorical data are expressed as absolute numbers and percentages. Non-normally distributed variables were analysed via the Wilcox rank-sum test, with confidence intervals calculated automatically using the “Stats” package in R [10]. The Kruskall–Wallis test was applied to the three groups of non-normally distributed variables, with confidence intervals determined using the bootstrapping method with the “Boot” package in R [11].

## 4 Surgical procedure

In cases of 44B1 fractures, a direct lateral approach to the lateral malleolus was obtained. Reduction was performed under fluoroscopy, and then the fracture was fixed with an anatomical plate. Finally, a hook test was performed under fluoroscopy, and if positive, the syndesmosis was stabilised with a quadri- or tricortical *trans*-syndesmotic screw.

In cases of 44B2 fracture, the previously described surgical approach was used, with a second, medial malleolus, access. Medial malleolus fractures underwent open reduction and fracture fixation with two screws or a plate, depending on the type of the fracture. The presence of a medial clear space greater than 5 ​mm on the mortise view indicated a deltoid ligament injury, and in such cases a 3.5-mm anchor was used.

In 44B3 fractures, the posterior malleolus was only surgically fixed in Bartonicek types II, III and IV. In such cases, the access was posterior-lateral, and open reduction and plate fixation of the posterior malleolus were performed. Through the same surgical approach, open reduction of lateral malleolus and fixation with plate and screw were performed. In addition, a second medial malleolus access was obtained, and the fracture was fixed with two screws or a plate, depending on the type of the fracture.

## 5 Results

**Fig. 1:**
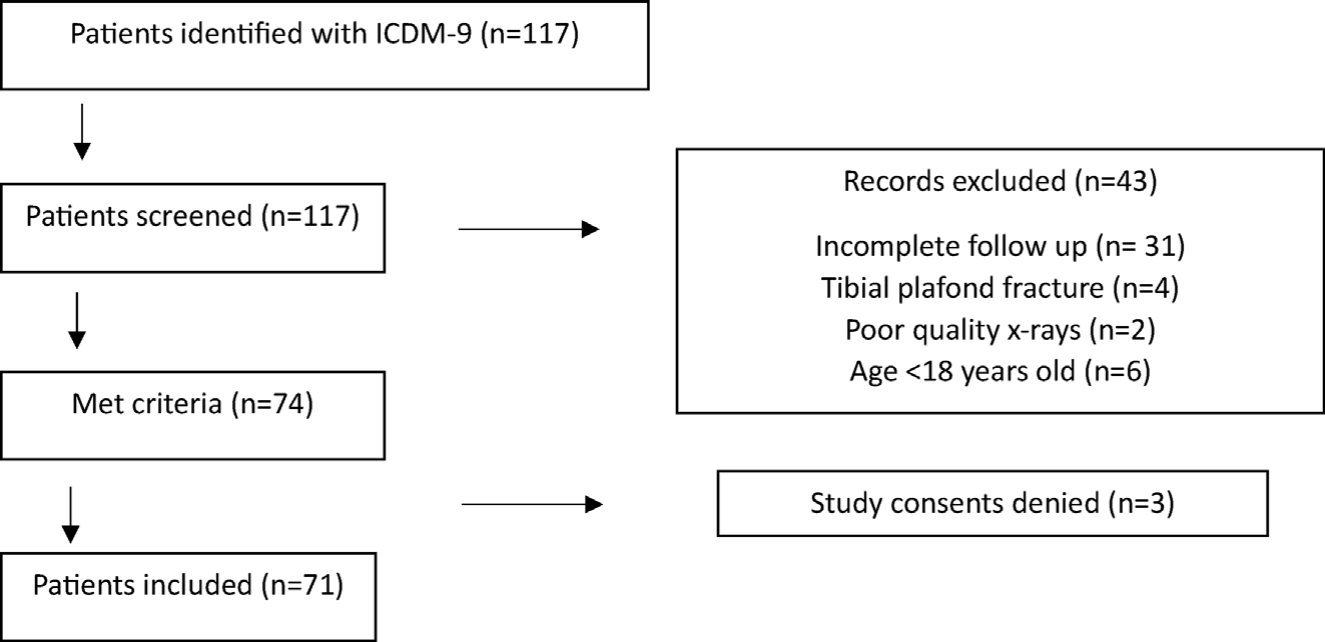
Study flowchart.

Of the 117 patients examined in the study, 71 meet the inclusion criteria (Fig. 1). The mean age was 61 years ​± ​16.0, and the average follow-up was 93.36 ​± ​14.66 months. 23 (32.4%) patients were male and 48 (67.6%) were female. Of the 71 patients who had a *trans*-syndesmotic AO/OTA 44B malleolar fracture, 26 (36.6%) were classified as 44B1, 30 (42.3%) 44B2 and 15 (21.1%) 44B3. The mean FADI score reported was 90.7 ​± ​9.9 (min. 41.3; max. 99). The ASA score was 2.5 ​± ​0.6 and the CCI score was 86.2 ​± ​14.8. The mean Body Mass Index (BMI) of the patient sample was 26.1 ​± ​4.2 (min. 17.5, max. 37.5). Twenty-nine (40.8%) were classed as having normal weight (BMI <25), 25 (35.2%) overweight (BMI between 25 and 30) and 17 (24%) obesity (BMI>30). 11 patients had performed heavy work with joint overload for at least 10 years. The mean K&L score was 2.25 ​± ​0.81. Thirty (42.2%) patients presented with ankle fracture-dislocations. The average waiting time before surgery was 4.0 ​± ​3.2 days, with 40 (56.3%) undergoing surgery in the first 3 days after the injury. 36 (50.7%) fractures were synthesized with syndesmotic transfixation. 21 (29.5%) were classed as having “poor joint reduction”, i.e., any postoperative step-off.

**Fig. 2:**
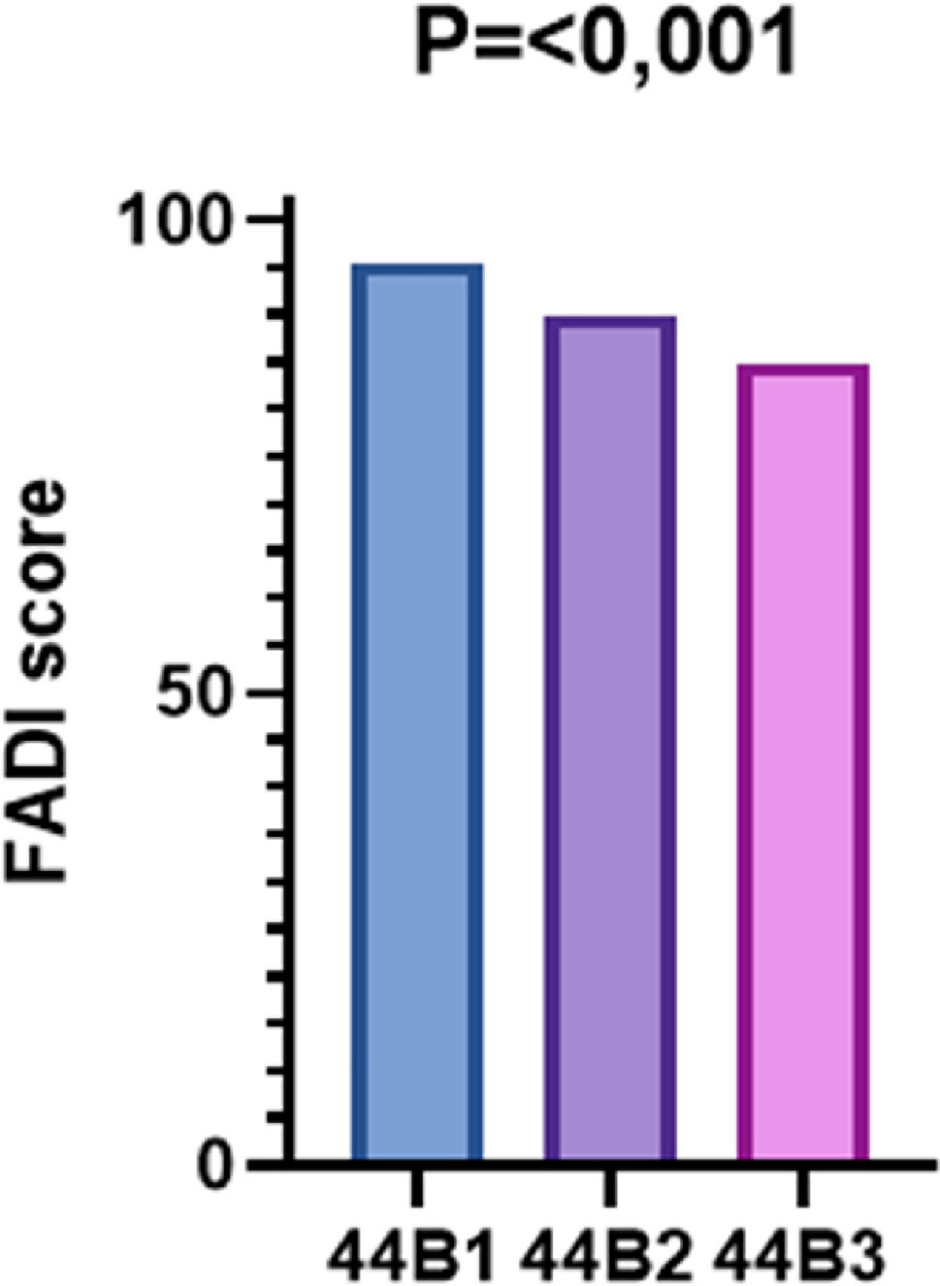
Relationship between AO/OTA classification and FADI.

The risk factors analysed in the study are summarized below (Table 1), alongside their respective p-values. Only those factors found to convey statistically significant risk are analysed in detail. Specifically, the FADI score in patients with AO/OTA 44B1 fracture was 95.5 ​± ​7.5, in 44B2 it was 90.0 ​± ​8.4 and in 44B3 it was 84.0 ​± ​13.0 (p ​< ​0.001 95% I.C. 8.39–35.16) (Fig. 2). In patients with BMI <25, it was 93.7 ​± ​6.7, while in those with BMI >25 it was 88.6 ​± ​11.4 (p ​= ​0.047 95%I.C. 0.01–8.10).

**Table 1:** The risk factors analysed with their respective p-values and 95% C.I.

Risk Factors	FADI score (p-value)	K&L Score (p-value)
Type of fracture	<0.001 (95% I.C. 8.39–35.16)	n.s.
Fracture/dislocation	0.002 (95% I.C. 0.01–8.9)	0.006 (95% I.C 0.01–1.00)
BMI	0.047 (95%I.C. 0.01–8.10)	n.s.
Intersyndesmotic screw	n.s.	n.s.
Heavy work	n.s.	n.s.
Surgery beyond 3 days	n.s.	n.s.
History of ankle sprains	n.s.	n.s.
Sex	n.s.	n.s.
Poor joint reduction	0.012 (95% I.C. 0.90–10.60)	0.012 (95%I.C. 0.01–1.00)

In patients without ankle fracture-dislocation the FADI score was 94.4 ​± ​6.0, while in patients with ankle fracture-dislocation it was 85.8 ​± ​11.98 (P ​= ​0.002 95% I.C. 0.01–8.9). In the former group, the K&L score was 1.97 ​± ​0.65, while in the latter it was 2.63 ​± ​0.85 (P ​= ​0.006 95% I.C 0.01–1.00).

In patients with good reduction quality, the FADI score was 93.42 ​± ​6.5, whereas in those with poor joint reduction quality it was 85.30 ​± ​13.5 (P ​= ​0.012 95% I.C. 0.90–10.60). In patients with good reduction quality the K&L score was 2.12 ​± ​0.68, but in patients with poor joint reduction quality it was 2.71 ​± ​0.93 (P ​= ​0.012 95%I.C. 0.01–1.00).

**Fig. 3:**
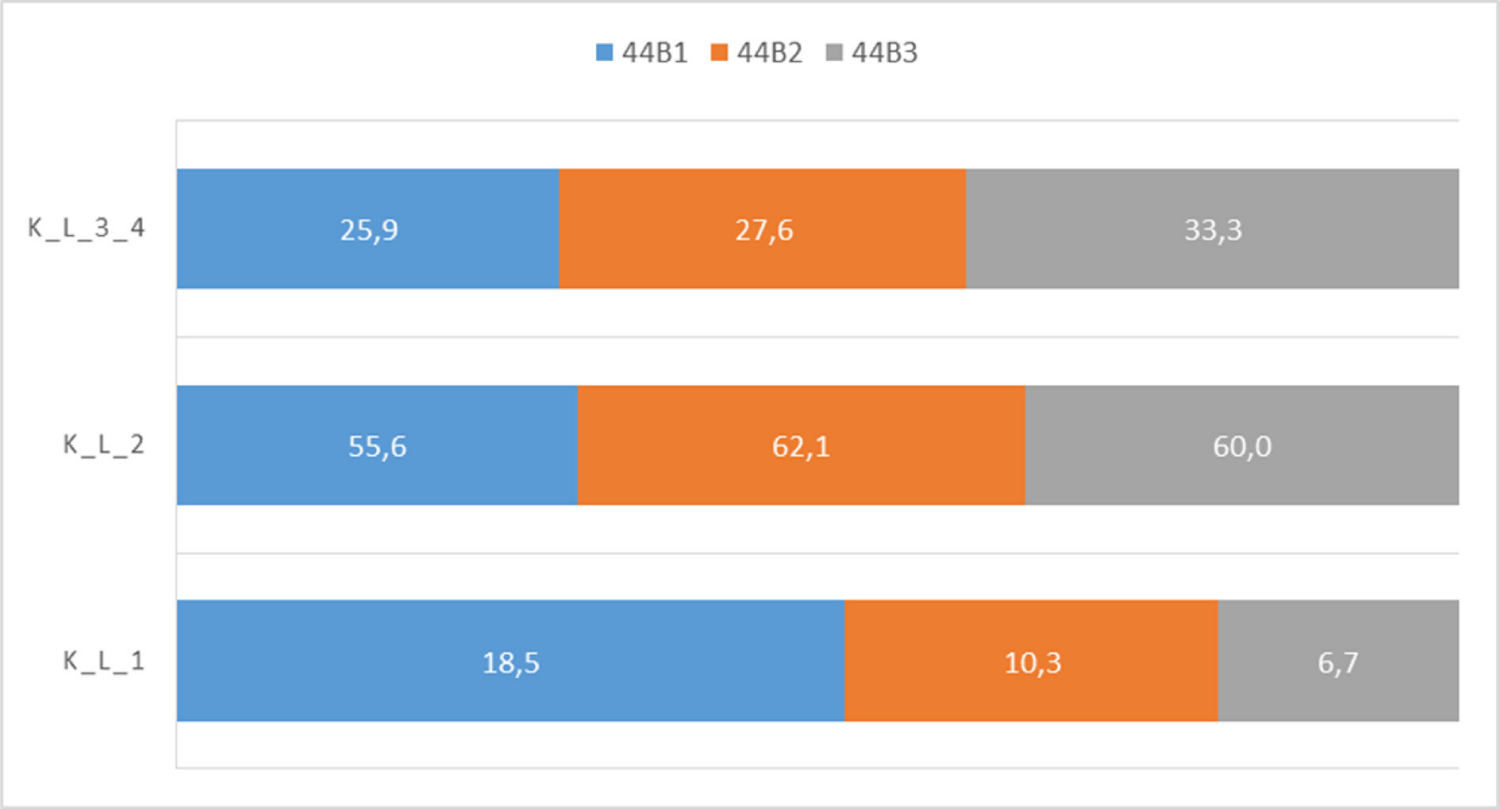
Relationship between AO/OTA classification and K&L.

Advanced osteoarthritis (K&L score 3–4) was found in 25.9% of patients with AO/OTA 44B1 ankle fracture, 27.6% of patients with AO/OTA 44B2 and 33.3% of patients with AO/OTA 44B3 (Fig. 3).

**Fig. 4:**
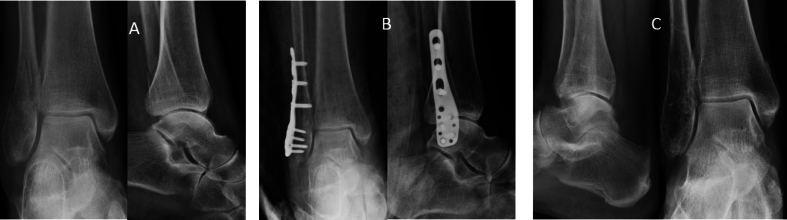
A 44B1 ankle fracture. A: pre-operative x-rays. B: after surgery x-rays. C: seven years follow-up x-rays.

**Fig. 5:**
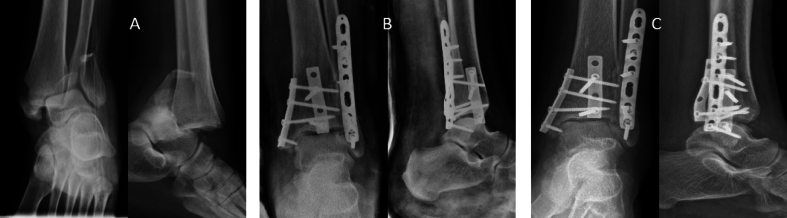
A case of 44B3 ankle fracture with an AO at the end of follow-up. A: pre-operative x-rays. B: after surgery x-rays. C: seven years follow-up x-rays. Last images show a reduction of joint space and sclerosis of the subchondral bone.

**Fig. 6:**
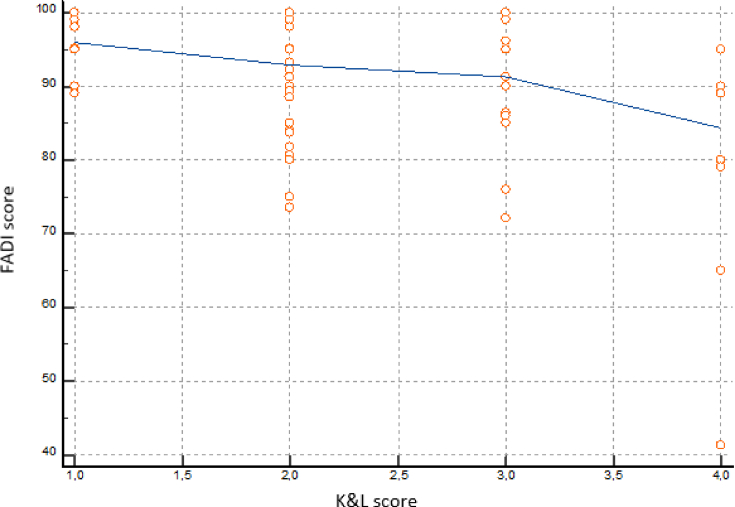
Correlation between K&L and FADI scores.

The relationship between the FADI score and the K&L score was analysed to assess whether a worsening of the patients' clinical outcome corresponded to a worsening of the radiographic picture (Fig. 4, Fig. 5). Analysis showed a linear relationship between the outcomes of the two clinical-radiographic scores, with a coefficient of −0.4403 and a 95% confidence interval ranging from −0.6109 to −0.2307 (P ​< ​0.001). This linear relationship reveals a worsening of the clinical outcome (lower FADI score) as the K&L score, i.e., the severity of the AO, increases (Fig. 6).

## 6 Discussion

This study investigated possible risk factors and predisposing factors to the onset of osteoarthritis at the talocrural joint fracture. Only AO/OTA 44B fractures were considered, since they are the most frequent and have high arthrotic potential [12]. To further streamline the analysis, we focused on a specific pattern of malleolar fracture, and therefore the same traumatic mechanism, characterized by an injurious force that exerts an axial load on the supinated foot.

The risk factors analysed can be divided into 3 categories: patient-related, fracture-related and surgical treatment-related. Among the patient-related risk factors, the most influential was BMI. BMI >25 was statistically correlated with functional worsening of the talocrural joint. In other words, patients with overweight or obesity tend to have worse clinical outcomes than patients with normal weight. This aligns with previous reports that obesity places patients at an increased risk of complex ankle fracture [13], and that patients with obesity experience increased pain and worse joint function due to chronic mechanical overload of the ankle [14].

Among the fracture-related factors, the severity of the injury was correlated with worse clinical outcome. Specifically, trimalleolar fractures had the most unfavourable clinical outcome, while patients with malleolar fractures with medial compartment involvement (AO/OTA 44B2), along with those with fibula injury associated with the medial and posterior malleolus (AO/OTA 44B3), reported higher FADI scores than those with malleolar fractures involving only the fibula (AO/OTA 44B1).

According to the literature, bimalleolar and trimalleolar fractures result in worse gait cycle as compared to peroneal malleolar fractures [14], and the presence of a malleolar fracture associated with medial malleolus fracture leads to an increased risk of developing AO [4,15]. A review by Stufkens et al. of data pertaining to a total of 1882 patients treated surgically for talocrural fractures found that AO/OTA 44B fractures results in a worse functional outcome over an average follow-up of 5 years [16].

According to our results, one of the most influential risk factors is fracture-dislocation, which is related to the onset of early AO [17]. Ankle fracture-dislocation comports a significantly higher risk of developing osteochondral lesions, leading to earlier AO [18].

A well-known intervention-related risk factor is reduction quality. As expected, patients in our sample with poor quality of reduction had worse clinical and radiographic outcomes. This is in line with a report that intra-articular step-off increases the peak contact stress by up to 300% [19]. Incorrect transmission of forces through the joint leads to early arthritis.

The instability of syndesmosis is also risk factor for osteoarthritis. As previously reported, ankle fractures with syndesmotic stabilization are associated with a high rate of secondary osteoarthritis related to the severity of the injury [20]. In our study, however, the intersyndesmotic stabilization was not a statistically significant factor due to the greater homogeneity of the sample under investigation.

Numerous radiographic scores are used to evaluate AO. However, the K&L score has good inter- and intra-observer reliability [21]. The patients analysed here presented a mean K&L score of 2.25 ​± ​0.81, indicating mild arthrosis characterized by slight joint space reduction. Twenty patients (28.1%) displayed advanced osteoarthritis, represented by a K&L score of 3 or 4.

Statistical analysis showed a linear relationship between the K&L score and FADI score outcomes. AO/OTA 44B2 and AO/OTA 44B3 *trans*-syndesmotic malleolar fractures were associated with a higher mean K&L score than AO/OTA 44B1 fractures. However, the lack of statistically significant data could be related to the need for longer follow-up. Indeed, a study by Horisberger M et al. conducted on 257 patients with advanced AO reported a mean latency of 21 years from the malleolar fracture to the finding of advanced osteoarthritis [22].

The study was limited by the systematic bias associated with retrospective studies. Furthermore, bias could have been introduced by human error in calculating FADI and K&L scores. Nevertheless, to our knowledge, this is one of the few articles to analyse risk factors in a single fracture pattern. This is important because it provides a better understanding of which risk factors are likely to influence outcomes in this particular type of ankle fracture.

## 7 Conclusion

The most influential risk factors for worse outcome in AO/OTA 44B ankle fractures were found to be BMI, injury severity, fracture-dislocation and reduction quality. However, further studies with longer follow-up are needed to assess their respective effects on AO evolution.

## Ethics approval

The study was approved by the local University-Hospital Human Subject Research Ethics Committee (Comitato Etico Indipendente di Area Vasta Emilia Centro—CE-AVEC 403/2022/Oss/AOUFe May 19, 2022).

## Consent to participate

Data collection and analysis was performed in compliance with the Declaration of Helsinki.

## Informed consent

Informed consent to participate was obtained for as many patients enrolled in the retrospective study as possible. Data is available upon request.

## Contributions

All authors have made substantial contributions to all of the following:

(1) The study concept and design, data acquisition, or data analysis and interpretation.
(2) Drafting the article or revising it critically for important intellectual content.
(3) Final approval of the version to be submitted.

## Funding

Open access funding provided by Università degli Studi di Ferrara within the CRUI-CARE Agreement.

## Studies involving humans or animals

Clinical trials or other experimentation on humans must be in accordance with the ethical standards of the responsible committee on human experimentation (institutional and national) *and* with the Helsinki Declaration of 1975, as revised in 2000. Randomized controlled trials should follow the Consolidated Standards of Reporting Trials (CONSORT) guidelines and be registered in a public trials registry.

Studies involving experiments with animals were in accordance with institution guidelines.

## Declaration of competing interest

The authors have no relevant financial or nonfinancial interests to disclose.
